# Prevention and Treatment of Life-Threatening COVID-19 May Be Possible with Oxygen Treatment

**DOI:** 10.3390/life12050754

**Published:** 2022-05-19

**Authors:** Jukka Ylikoski, Jarmo Lehtimäki, Rauno Pääkkönen, Antti Mäkitie

**Affiliations:** 1 Department of Otorhinolaryngology—Head and Neck Surgery, University of Helsinki and Helsinki University Hospital, 00029 Helsinki, Finland; jukka.ylikoski@fimnet.fi (J.Y.); rauno.paakkonen@ains.fi (R.P.); 2 Helsinki Ear Institute, 00420 Helsinki, Finland; jarmo.lehtimaki@gmail.com; 3 Salustim Group Inc., 90440 Kempele, Finland

**Keywords:** SARS CoV-2, hyperbaric oxygen, autonomous nerve system, brain hypoxia, dysautonomia

## Abstract

Most SARS CoV-2 infections probably occur unnoticed or cause only cause a mild common cold that does not require medical intervention. A significant proportion of more severe cases is characterized by early neurological symptoms such as headache, fatigue, and impaired consciousness, including respiratory distress. These symptoms suggest hypoxia, specifically affecting the brain. The condition is best explained by primary replication of the virus in the nasal respiratory and/or the olfactory epithelia, followed by an invasion of the virus into the central nervous system, including the respiratory centers, either along a transneural route, through disruption of the blood-brain barrier, or both. In patients, presenting with early dyspnea, the primary goal of therapy should be the reversal of brain hypoxia as efficiently as possible. The first approach should be intermittent treatment with 100% oxygen using a tight oronasal mask or a hood. If this does not help within a few hours, an enclosure is needed to increase the ambient pressure. This management approach is well established in the hypoxia-related diseases in diving and aerospace medicine and preserves the patient’s spontaneous breathing. Preliminary research evidence indicates that even a small elevation of the ambient pressure might be lifesaving. Other neurological symptoms, presenting particularly in long COVID-19, suggest imbalance of the autonomous nervous system, i.e., dysautonomia. These patients could benefit from vagal nerve stimulation.

## 1. Introduction

Many countries are still struggling with COVID-19. The disease has caused many fatalities because no effective treatment has been available. At the beginning of the COVID-19 pandemic, the causative agent, SARS-CoV-2, was found to show great similarities with 2002/3 SARS-CoV and 2012 MERS-CoV. Consequently, severe COVID-19 was primarily regarded as a pulmonary one-organ disease, with pneumonia and bronchiolitis leading to dyspnea, and in the most severe cases to acute respiratory distress syndrome (ARDS) and septic shock. The SARS-CoV-2 virus was thought, in severe cases, to affect part of the innate immune response and to activate an inflammatory cascade stimulating the release of cytokines and chemokines, particularly within the lungs [1,2,3]. This would lead to a robust inflammatory response that, if it was not controlled, could result in a “cytokine storm” with detrimental systemic consequences [3].

However, even from the beginning of the pandemic, COVID-19 surprised by somewhat commonly causing a wide spectrum of clearly extrapulmonary symptoms that do not fit the aforementioned pulmonary concept. There were several early reports of patients with COVID-19 seeking medical attention who presented themselves with pure neurological manifestations at onset with nonneurological features first manifesting days later. It was proposed to term these cases “neuro-COVID syndrome” [4].

Among these early symptoms were impaired consciousness, fatigue, and headache, which all pointed to impaired oxygenation in the central nervous system (CNS). These usually appeared in combination with respiratory distress as the usual cause for hospitalization. We and others have stated that these symptoms are best explained by early neuroinvasion by SARS-CoV-2 of the CNS, including respiratory centers, leading to brain hypoxia [5,6,7]. In the first report consisting of 214 hospitalized severely affected patients, during January–February 2020 in Wuhan, 36% had neurological manifestations, and in some, these were the only symptoms. Impaired consciousness was seen in 15% of the cases, and it occurred early in the illness, i.e., during the first 1–2 days [5]. It is obvious that in such cases the primary target of therapy should be brain hypoxia and not a pulmonary disease. Therefore, the prevailing global principle in anesthesiology concerning oxygenation therapy as distinct from oxygen treatment, i.e., “as little oxygen as possible”, might not be accurate for hypoxic COVID-19 patients. Based on our background experience in diving, aviation, and other potentially hypoxia-generating conditions, we have analyzed the reports of the clinical course, including signs and symptoms, of COVID-19. Our analyses have revealed that most of the early neurological symptoms of COVID-19 resemble those of mild brain hypoxia. Therefore, we propose that it is the oxygen deficiency in tissues, particularly in the brain, that should be the main target of therapies.

## 2. Potential Pathophysiological Mechanisms of COVID-19 

### 2.1. Early Neurological (Extrapulmonary) Symptoms

Initially, COVID-19 was predominantly characterized as a respiratory illness targeting the upper airways, similar to other human coronaviruses. Clinical findings in people infected with SARS-CoV-2 range from an asymptomatic course to severe pneumonia requiring mechanical ventilation. The pathophysiology and severity of COVID-19 illness vary among patients and depend in part on underlying risk factors and chronic diseases. Its pathogenesis follows that of other respiratory viruses typically replicating in the epithelia of the nasal cavity or nasopharynx. Even from the early reports from Wuhan, it was clear that the great majority of COVID-19 cases show only symptoms of a mild, common cold-type upper respiratory infection [8] and recover within one or two weeks. However, these reports also described a significant proportion of hospitalized patients and even about 25% of severe cases presenting with neurological symptoms, including anosmia, impaired consciousness, and dyspnea, but normal pulmonary CT scans [8,9]. It was reported that most (up to 89%) of COVID-19 patients admitted to the intensive care unit could not breathe spontaneously [9]. There were also reports of COVID-19 patients presenting with asymptomatic (silent) hypoxia in whom their early respiratory dysfunction was described as a “cessation of spontaneous breathing.” These features suggested a central apnea or failure in the feedback loop from the pulmonary receptors to the respiratory centers, mediated by the vagus nerve [10]. Note that hypoxia may also result from a combination of these two. 

So far, several pieces of evidence have clearly shown that SARS-CoV-2 affects not only the respiratory tract but also the CNS, resulting in dyspnea and clearly neurological symptoms such as loss of smell and taste, headache, fatigue, impaired consciousness, nausea, and vomiting in more than one third of individuals with COVID-19 [11,12]. Therefore, it was hypothesized, first from Wuhan and later on in many other reports, that SARS-CoV-2 can be neurotropic, entering the body through the nose and spreading to the CNS, including the respiratory centers in the brain stem and medulla. Accordingly, the most dangerous symptom, the initial respiratory failure, would be neurogenic in origin [4,5,6,7,13,14]. Note that even in the early reports on COVID-19, impaired consciousness, in some patients the only symptom, was described as occurring in 15–50% of cases [5,6,15,16]. It is particularly noteworthy that most neurological manifestations could occur very early in the illness.

**Figure 1:**
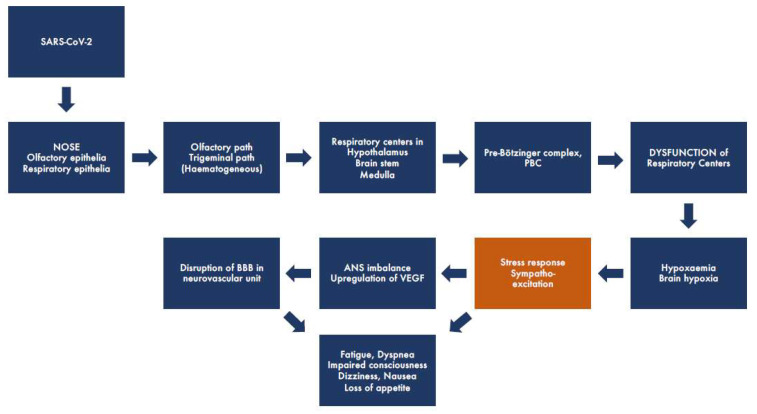
Schematic representation showing the putative brain pathway of SARS-CoV2 in COVID-19. The virus enters the respiratory or olfactory epithelia of the nasal cavity, spreads (by trans-synaptic migration) to the brain through the olfactory or trigeminal tracts, and infects deeper parts of the brain, including respiratory centers in the thalamus, brain stem, and medulla. This leads to dysfunction of the respiratory centers, including Pre-Bötzinger complex, causing respiratory distress and hypoxaemia/hypoxia and leading to a strong stress response associated with sympathoexcitation. Dysfunction of the ANS triggers upregulation of VEGF and disruption of the BBB, further leading to the worsening of hypoxia, resulting in both acute and long-standing neurological symptoms.

From accumulated human and experimental research data, we have constructed the putative, simplified pathogenetic pathway of SARS-CoV-2 (Figure 1). It seems clear that the virus enters the body by first attacking and replicating in the respiratory or olfactory epithelia of the nasal cavity and causing a common cold-like upper respiratory infection, with the highest viral replication occurring in the nose at day four [17]. Olfactory engagement seems apparent from the common occurrence of anosmia that has been described as occurring in more than 90% of cases seeking medical attention [9,18]. The olfactory epithelium contains, in addition to sustentacular and microvillar cells, olfactory sensory neurons. Their peripheral olfactory cilia are protected from the external air by only a thin mucous blanket. Therefore, the olfactory nerve has been described as a shortcut into the CNS for several viral diseases [19]. The SARS-CoV-2 spike (S)-protein that binds to its specific receptor ACE2, in concert with host proteases—principally TMPRSS2 (promotes cellular entry)—is co-expressed in epithelial type II pneumocytes in the lungs and in ciliated and goblet cells of the nasal epithelium [20]. Olfactory epithelia as the entry site of SARS-CoV-2 into the CNS were strongly supported by a recent autopsy material study of 33 COVID-19 victims. By using immunohistochemistry, in situ hybridization, and electron microscopy, it was possible to visualize the presence of intact CoV particles together with SARS-CoV-2 RNA in the olfactory mucosa [21]. That study also revealed viral particles and RNA in neuroanatomical areas receiving olfactory tract projections that may suggest SARS-CoV-2 neuroinvasion into deeper parts of the brain, including respiratory centers in the thalamus and brain stem, through axonal transport. This is not unexpected, as human coronaviruses have been shown to be potentially neurotropic and induce immune overactivation [22]. Another possible path for CNS spread is the hematogenous route, which involves early viral crossing of the blood-brain barrier (BBB). In general, the effect of COVID-19 on the brain may take several forms, some via direct infection and others via secondary mechanisms, e.g., immune response or respiratory failure-induced hypoxia. Direct presence of SARS-CoV-2 in the brain has been demonstrated through the detection of SARS-CoV-2 RNA in the cerebrospinal fluid of infected patients [23]. 

Regarding the extent of SARS-CoV-2 being able to affect the brainstem, it has been hypothesized that the respiratory breakdown in COVID-19 patients may be caused at least in part by SARS-CoV-2 infecting and destroying respiratory centers in the medulla oblongata and the pons [14]. Two sets of neuronal networks within the brainstem are crucial to the generation of respiratory rhythm, the Pre-Bötzinger complex (PBC), the pacemaker of the respiratory rhythm generator—also proposed as the kernel of respiration—and the retrotrapezoid nucleus/parafacial respiratory group [24]. When, the PBC was shut down in a mouse model of SARS CoV, it caused lethality due to respiratory failure [25]. It was hypothesized that SARS CoV-2 behaves similarly and that the destruction of the respiratory center (PBC) in the brainstem could be accountable for respiratory breakdown in COVID-19 patients [9,14].

It has also been suggested that the virus might enter the lowest region of the brain stem, the dorsal vagal complex (DVC), located in the medulla oblongata, which is involved in the control of several autonomic activities including breathing, food intake, nausea, and vomiting: these are all frequent extrapulmonary symptoms of COVID-19 [26]. In the DVC, the nucleus of tractus solitarius (NTS) is known, in addition to the hypothalamus, to be involved in food intake. The loss of appetite, sometimes a prominent symptom in COVID-19, means that the crosstalk between the hypothalamus and DVC has been broken as in pathological states such as under stress [27]. It is well established that another component of the DVC, the area postrema lying beneath the fourth cerebral ventricle, plays a crucial role in the elicitation of nausea and vomiting. This structure, together with DVC, NTS, and the dorsal motor nucleus of the vagus (DMNV), forms neurocircuits that have been classified as the “emetic chemoreceptor trigger zone” [28]. Interestingly, the eventual involvement of the subnucleus of NTS, the gelatinous nucleus, has been documented in the respiratory failure of sudden infant death syndrome (SIDS). [27]. Taken together, this neuroinvasive propensity leading to respiratory dysfunction has been demonstrated as a common feature of CoVs. Furthermore, hypoxemia and subsequent hypoxia are known to induce a stress reaction with the imbalance of the ANS and in the CNS, particularly of the central autonomic network (CAN) [29]. Hypoxia itself, or in combination with ANS/CAN imbalance, is a well-known cause of BBB disruption, leading to a multitude of local and systemic manifestations (Figure 1).

### 2.2. Significance of Virus Genotypes for Virulence Symptoms and Neurotropism

A critical initial step of infection is the interaction of the virus with receptors on host cells. The target tissues for viral infection, i.e., tissue tropism, is determined by the availability of virus receptors and entry cofactors on the surfaces of host cells. In the case of SARS-CoV-2 and other coronaviruses, the receptor binding occurs through the spike (S) protein on the virus surface. Both SARS-CoV-2 and the related SARS-CoV bind to ACE2 on human cells. ACE2, however, is expressed at low protein levels in respiratory and olfactory epithelial cells [30]. Although previous analyses have revealed that TMPRSS2, the primary protease important for SARS-CoV-2 entry, is highly expressed in different tissues, it was presumed that additional cofactors are required to facilitate virus-host cell interactions in cells with low ACE2 expression. It was shown that the membrane protein neuropilin-1 (NRP1) promotes SARS-CoV-2 entry by interacting with the SARS-CoV-2 S-protein and that NRP1 could thus represent an ACE2 potentiating factor by promoting the interaction of the virus with ACE2 [31,32]. 

It has been presumed that the pathogenic pathways and high transmission potentials of human coronaviruses are facilitated by an interplay between epigenetics and coronavirus infection. SARS-CoV-2 utilizes multiple ways for cellular entry (both nonendosomal and endosomal) and potentially uses various means of epigenetic control to inhibit the initiation of the host innate immune response. During the course of the pandemic, this virus efficiently has undergone genomic rearrangements, thereby developing important means for immunological escape. Such mutations have been especially effectively revealed by performing genome analyses of the SARS-CoV-2 sequences over the course of the COVID-19 pandemic in Costa Rica with a population of five million. Those analyses reveal the detection of mutations in line with other studies but also point out the local increases, particularly in the detection of Spike-T1117I variant. These constant genomic rearrangements may offer some explanation for the variations in transmission rates, symptoms, tropisms, and virulence of the SARS-CoV-2 [33,34,35].

### 2.3. The Role of the Blood-Brain Barrier

It is well-known that the BBB is a dynamic platform, collectively referred to as the neurovascular unit (NVU), responsible for the exchange of substances between the blood and the brain parenchyma and that it is an essential functional gatekeeper for the CNS. The propensity less commonly attributed to the BBB is its responsibility for the exchange of oxygen, which plays a critical role in the maintenance of brain homeostasis. Dysfunction of the BBB/NVU is a characteristic of several neurovascular pathologies. Moreover, physiological changes, environmental factors, nutritional habits, and psychological stress can modulate the tightness of the barrier. Mild inflammation is often associated with reduced BBB integrity, as observed for instance in obesity or psychosocial stress. Among extrinsic insults known to induce BBB breakdown, hypoxia is probably the most well characterized, but many knowledge gaps remain. Hypoxia can disrupt the BBB and result in increased permeability, vasogenic edema, and tissue damage [36]. Of the different types of hypoxia, hypobaric hypoxia (HH) is probably the best characterized because under hypobaric conditions, during ascent, it possible to monitor the gradual progression of HH. The proposed mechanisms for NVU damage by HH, and hypoxia in general, include induction of increased expression hypoxia-inducible factor-1 (Hif-1), enhanced endothelial transcytosis and oxidative stress [37]. It is well known that increased Hif-1 leads to increased expression of vascular endothelial growth factor (VEGF) in activated astrocytes, which further leads to NVU damage through changes in its tight junctions. One major mechanism associated with hypoxia-induced BBB opening is enhanced endothelial transcytosis that may be mediated by factors such as nitric oxide, calcium influx, or the release of inflammatory cytokines [38]. This may be a major mechanism because different cellular components of NVU show distinct differences in sensitivity to oxygen deprivation, so that endothelial cells (ECs) are markedly more sensitive than are pericytes or astrocytes. It is currently unclear whether the neurological symptoms in COVID-19 are a direct result of neural infection or secondary to endothelial cell infection, hypoxia, or circulating pro-inflammatory cytokines.

Several reports bring up the hypothesis that COVID-19, because it produces protean manifestations ranging from head to toe, represents an endothelial disease [39]. A similar pathophysiological mechanism has been proposed for long COVID-19. That hypothesis states that the initial pathology is due to the virus binding to the ACE-2 protein on ECs lining blood vessels and entering these cells in order to replicate, in turn releasing the immune response and thereby symptoms [40]. It even states that after initiating this immunologic cascade, the nascent virus spreads further into the nasopharyngeal tract. The early effect on the EC system is particularly attractive because it could explain, through dysregulation of the BBB and further dysautonomia, the relatively commonly occurring neurological symptoms of long COVID-19. The early EC effect by SARS-CoV-2, however, seems to be a rarity because virus RNA has usually not been found in the blood of patients with early COVID-19 [17,41,42,43]. However, several studies have found SARS-CoV-2 viremia and as such strong support for virus brain entry across the BBB at later stages of severe COVID-19 [44,45,46,47]. 

### 2.4. Role of Autonomic Dysfunction in Symptoms of COVID-19 and Long COVID-19

All our unconscious bodily functions are controlled by the ANS, and particularly by the CAN [29]. The most common cause for the dysfunction of the CAN is stress, the major cause of deteriorating health conditions and illnesses. It is well-known that infections, including viral ones, are associated with oxidative stress and subsequent reactive oxygen species. Recent research, by investigating small RNAs, powerful stress markers in the blood samples of patients with moderate or severe COVID-19, has revealed that the cells of COVID-19 patients undergo tremendous stress [47].

The CAN initially reacts to stressor effects with a sympathetic fight/flight response that is restored to normal by the parasympathetic nervous system’s relax/digest response [48]. Many symptoms and illnesses result from the inability of the parasympathetic activity to restore the ANS balance (for review see [49]). These two circuits, the sympathetic and parasympathetic systems, are constantly interacting by heart rate variability (HRV) as a read-out of ANS balance; thus, HRV may consequently serve as a measure of stress [50].

The vagal system, with the vagus nerve terminating at the DVC in the brain stem, is known to be the key factor in most aspects of respiration. It both transmits the sensory information from pulmonary chemoreceptors to the CNS and is responsible for the activity that provides appropriate stimuli to the nuclei of the respiratory centers of the brain stem. This activity includes stimuli to NTS and nucleus ambiguus to give efferent commands to the respiratory muscles to function effectively. Interestingly, in some cases, the initial respiratory failure in COVID-19 can be a weakness or paresis of the diaphragm muscle, which is one of the two pumps necessary for life. Phrenic nerve paralysis has been described in a COVID-19 patient [51]. The paralysis of the diaphragm muscle was the cause of respiratory failure in connection with bulbar polio virus. Notably, the symptoms of “long polio”, which can occur as long as 15 years after the acute stage, resemble those of long COVID-19 (fatigue, headache, musculoskeletal pains) [52].

Since the COVID-19 pandemic began, there has been a concern that survivors might be at an increased risk of neurological disorders. This concern, initially based on findings from infections with other coronaviruses, was rapidly followed by case series studies of the current pandemic. Multiple studies reported on the long-term outcomes of SARS survivors in Toronto in 2003. Most patients had persistent functional disabilities and were unable to return to their work. Their persisting debilitating physical symptoms included musculoskeletal pains, profound fatigability, shortness of breath, psychological distress, and major sleep problems [53,54]. These neurological long-term sequelae strongly suggest that they had been caused by an infection or inflammation in the CNS as a causative factor. 

Following the initial surge of infections by SARS-CoV-2, focus has shifted to managing the longer-term sequelae of illness survivors. Post-acute COVID-19 syndrome (known colloquially as long COVID-19) is emerging as a prevalent syndrome. It encompasses a plethora of debilitating symptoms (including fatigue, breathlessness, pain, palpitations, sleep disturbance, and cognitive impairment (“brain fog”), which can last for weeks or months following the acute stage, even after a mild illness [55,56]. These symptoms thus greatly resemble those seen in follow-up studies of SARS2002/3 survivors [53,54]. As in the case of SARS-CoV, these neurological long-term sequelae of SARS-CoV-2 strongly suggest that their cause is an infection or inflammation in the CNS, not only in the lungs. Most patients recover from COVID-19 within a few weeks, but in surprisingly many, the (neurological) symptoms can continue for a long time [55,56]. These symptoms refer to imbalance in the CAN (with sympathetic dominance), “dysautonomia.” This dysautonomia is supposed to be caused by cerebral hypoperfusion that leads to an overactive sympathetic system (fight or flight) with correspondingly reduced parasympathetic (relax) activity [57].

## 3. Therapies of COVID-19 and Long COVID-19

Most SARS CoV-2 infections probably occur unnoticed or cause only mild common cold symptoms that need no treatment. Pneumonia is probably the most common complication, but in the same way as ARDS occurs at later stages and thus cannot be responsible for early neurological symptoms such as impaired consciousness beginning during the first days of the disease, sometimes without any other symptoms.

It is well established that COVID-19 currently lacks curative therapy. However, the above-described mechanisms of the early dyspnea and hypoxia being due to a dysfunction of the central regulatory mechanisms of respiration shift the main focus of therapeutic efforts to the stage of early brain hypoxia. This pathophysiological concept suggests that therapeutically, the most important step would be to correct the hypoxemia and subsequent brain hypoxia by optimizing the efficacy of oxygen treatment in preserving the spontaneous breathing in affected individuals. 

The role of the ANS, and particularly its CAN partition, in the disease process of COVID-19 appears to be crucial from the first symptoms to the final stage. Therefore, returning autonomic imbalance to normal might be important as a part of the therapeutic regimen, particularly in the patient population suffering from long COVID-19.

## 4. Oxygen Treatment

### 4.1. Principles of the Delivery of Oxygen into the Body and Its Administration

All of the body’s tissues rely on a continuous oxygen supply at a rate that matches their changing metabolic demands. The amount of dissolved oxygen within the tissues and the cells depends crucially on the atmospheric partial pressure of oxygen and how effectively respiration is able to deliver oxygen from air to the blood plasma. Partial pressure of oxygen (PO_2_) depends only on the atmosphere’s barometric pressure (BP), and at normal BP conditions the content of inspired oxygen is 20.8%. The oxygen delivery chain begins in the nose, where the inspired air is warmed, humidified, and delivered by convection through the trachea to the lung alveoli and further to circulation, with the destination being the mitochondria (Table 1).

**Figure 2:**
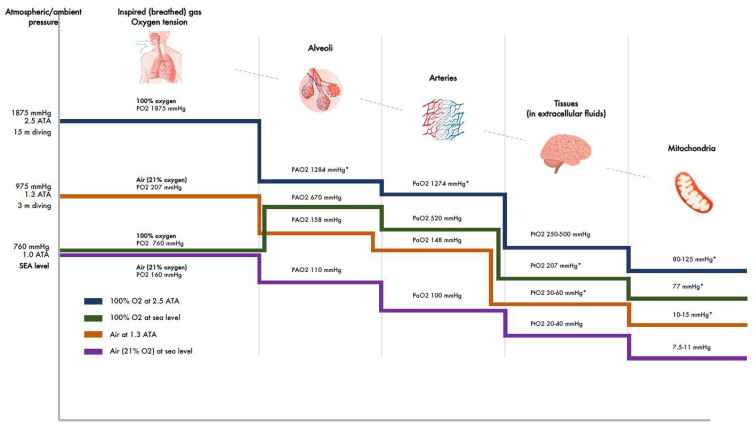
Oxygen tensions in the body. Reduction of oxygen tensions at different levels of airways, arteries, and tissues after breathing air at sea level and 1.3 ATA and breathing 100% oxygen at sea level and 2.5 ATA. (ATA = atmospheres absolute; PO_2_ = partial pressure of oxygen; PAO_2_ = partial pressure of oxygen in alveoli; PaO_2_ = partial pressure of oxygen in arteries; PtO_2_ = partial pressure of oxygen in tissues. PmO_2_ = partial pressure of oxygen in mitochondria. * Estimations through extrapolations).

At sea level in normal BP (760 mmHg, 1 ATA, atmospheric absolute), the PO_2_ of inspired air is 160 mmHg. Along its route down to the alveoli, the PO_2_ is reduced through various resistances, and final reduction takes place in alveoli due to dead space and the mixing of inspired and expired gases, resulting in a partial pressure of oxygen in alveoli (PAO_2_) of about 110 mmHg (Table 1; Figure 2). From the alveoli, oxygen diffuses across the alveolar-capillary membrane to pulmonary circulation with only a small reduction in partial pressure (PaO_2_ about 100 mmHg). From the pulmonary capillaries and arteries, the oxygen is carried to all parts of the body in two forms—a major fraction (up to 99%) that is bound to hemoglobin (Hb) and a small free fraction that dissolves in the plasma. Therefore, the number of red blood cells will dominantly affect the total capacity of oxygen delivery. However, at an elevated partial pressure of oxygen (such as during hyperbaric conditions), the dissolved amount can become significant. In all cases, the diffusion gradients are the oxygen’s driving force from the plasma to the mitochondria. Thus, the free dissolved fraction only is transported to mitochondria. While it is transferred to various tissues, the PaO_2_ is evenly reduced so that its level of extracellular tissue fluids (PtO_2_) at sea level is 20–40 mmHg. From there, PtO_2_ further reduces as oxygen diffuses to cells and mitochondria (partial pressure of oxygen 7.5–11 mmHg) (Table 1; Figure 2).

Note that the figures above apply to healthy young individuals in normal BP conditions. Factors that reduce PaO_2_ include age, humidification, and barometric changes. It has been shown that alveolar diffusion capacity decreases with aging, about 0.24 mmHg per year. Consequently, PaO_2_ in pulmonary capillaries in persons < 24 years is 99 mmHg, and 82–93 mmHg in those > 64 years (Table 1; Figure 2) [65]. Humidification in the nose will add of water vapor to inspired air, and its pressure is constant at 47 mmHg at normal body temperature (37 °C) [66]. This leads to a reduction of PAO_2_ by about 10 mmHg- and a corresponding reduction of PaO_2_. 

In oxygen therapy planning, it remains important to recognize that the pressure (PO_2_), and therefore the oxygen concentration, will determine the efficacy of oxygen treatment, i.e., how much oxygen will be transferred from the lung alveoli through the alveolar membrane to the arterial (capillary) blood. The quantity of dissolved oxygen in blood plasma, not the hemoglobin saturation, determines how much oxygen is diffused to the tissue. Further, according to Henry’s law, the pressure of oxygen determines the quantity of dissolved oxygen in blood plasma.

Currently, most COVID-19 patients who are hospitalized because of respiratory distress are treated according to modifications of guidelines originally published by the British Thoracic Society (BTS) in 2008 [67] and of the newer modification of these BTS guidelines constituting of oxygen delivery through high-flow nasal oxygen (HFNO) with an O_2_ flow of 40–60 L per minute [68]. This HFNO method, in addition to providing more O_2_, also necessitates the use of some elevated pressure, and thereby, lung alveoli are opened more efficiently. These guidelines stress the importance of the prevention of tissue hypoxia. However, this oxygen treatment strategy is targeted at correcting oxygen deficiency primarily in one tissue, blood (hypoxemia). Notably, blood is one of the tissues that is extremely resistant to oxygen deficiency, at least compared with brain tissue. 

Many patients undoubtedly benefit from the current treatment consisting of respiratory assistance and supplemental oxygen. However, if the tissue hypoxia is severe enough, this treatment may remain inadequate. This is because with employed extra-oxygen methods (including HFNO and loose masks), the content of inspired oxygen is only slightly increased. With a nasal cannula set at 2 L/min, oxygen tension of the inspired air ranges anywhere between 24% and 35%, leading only to a small increase in PaO_2_ [69].

This might be sufficient to prevent tissue hypoxia, but it may not be enough to correct it. The only means to effectively correct tissue hypoxia is to increase the content of plasma dissolved oxygen. This is possible by providing the patient’s alveoli with 100% oxygen by using a tight oronasal mask or hood, thereby by practicing conventional oxygen therapy, as for example with severe pulmonary damage [70]. Using this method, the plasma PaO_2_ can be increased about fivefold (from about 100 to 500 mmHg) [71] (Table 1; Figure 2). An appropriate dose could be 40–60 min of 100% oxygen given twice per day. If this does not help within a few hours, an enclosure is needed to increase the ambient pressure. Hypoxemia with COVID-19, as reported, is usually accompanied by an increased alveolar-to-arterial oxygen gradient, signifying either ventilation–perfusion mismatch or intrapulmonary shunting [72,73]. Preliminary experience from Wuhan suggests that HBOT may solve this problem [74].

Oxygen was first used as a specific treatment by John S. Haldane, often called the father of oxygen therapy, in Ypres, Belgium, for victims of chlorine gas attack in 1915. In the first instance of chemical warfare in history, the German forces bombarded the British front lines with 6000 pressurized bottles containing poisonous chloride gas. Thousands of young men died, while those who survived suffered from severe pulmonary damage. Haldane designed a tightly fitting oronasal mask, connected an oxygen bottle to it, and rescued a large number of victims with oxygenation therapy using 100% oxygen. The method developed by Haldane began to be used in other medical emergencies such as in CO poisoning and also in diving, aviation, and by mountaineers.

### 4.2. Oxygen Toxicity

Already Haldane was already very well aware that long-term 100% oxygen treatment can be accompanied by pulmonary damage and other adverse effects. He stressed that all therapies must be given in appropriated dosages and that the negative effects of hyperoxia may be avoided if hypoxia is confirmed before oxygen therapy is initiated. It is indisputable that long-term 100% oxygen breathing can cause pulmonary damage [75]. The mechanism is still uncertain, but the strongest candidate is the so-called absorption collapse theory. The normal maximal lung capacity is 3.5–4.5 L, but at rest we only breathe about 0.5 L and thus use only a small part of our lungs. The peripheral (“resting”) parts contain mainly nitrogen (78% of air) that keeps them open. Long-term oxygen breathing slowly leads to the replacement of nitrogen by oxygen in these pulmonary “resting areas”. The ensuing diffusion of oxygen through alveolar membranes into blood capillaries will lead to decrease in the alveolar gas pressure, and further, to their gas pressure vacuum and collapse (atelectasis or absorption collapse). This causes local hypoxia and subsequent further cellular damage with free radicals that invite inflammatory cells to the scene, and an infection arises. The problem can be easily avoided by taking air breaks in oxygen breathing, thereby filling the alveoli with nitrogen. Air breaks have been used in hyperbaric oxygenation therapy (HBOT) for decades, and this has diminished the myth of oxygen itself being toxic.

When, in 1977, in addition to pulmonary damage theory, a highly recognized biochemist named Irwin Fridovich [76] reported that oxygen therapy created free radicals, a general perception of oxygen toxicity arose. Although Fridovich admitted that he was wrong in another article two years later (1979) [77] and that it was not the oxygen but, on the contrary, hypoxia that creates free radicals, the general perception of oxygen toxicity has remained the generally accepted “fact.” That concept has continued to live a life of its own, particularly among anesthesiologists, to this day.

### 4.3. Oxygen Treatment under Increased Ambient Pressure

From the above, it can be concluded that the amount of blood PaO_2_ can be increased and thereby tissue hypoxia corrected by using 100% oxygen with a tight oronasal mask. However, a much more rapid and efficient method would involve elevating the ambient pressure at which oxygen is administered. A large amount of reliable human experimental knowledge is available, particularly from aviation and mountain climbing, about the importance of pressure for brain oxygenation. It was as early as in 1862 when the British hydrogen ballooners James Glaisher and Henry Coxwell described their experience in *Lancet* [78]. They climbed to 9000 m, where the content of oxygen remains at about 21% but the partial pressure falls because the atmospheric pressure is only one third of that at sea level. Glaisher suddenly lost consciousness, and all of Coxwell’s limbs became paralyzed. However, he was able to open the gas valve by drawing the valve rope with his teeth. Only after a descent of 50 m did their consciousness and bodily functions recover, and they survived. That was not the case for many other early ballooners who were supposed to have died of cold and brain hypoxia.

It seems clear that if brain hypoxia plays a role in the early extrapulmonary symptoms of COVID-19, it should be the primary target of treatment. Noninvasive brain oxygen state monitoring has been relatively reliably used with commercial near-infrared spectroscopy (NIRS) brain oximeters [for review see [79]]. Surprisingly, to the best of our knowledge, the use of NIRS on COVID-19 patients has not been reported so far. 

The significance of pressure elevation is easy to understand from the fact that by pressurizing air (20.8% oxygen) to such a low overpressure as 1.3 ATA (corresponding to 3 m diving), the partial pressure of oxygen (PaO_2_) in blood rises from 95 mmHg to 148 mmHg or 50% (Figure 2) [60]. This elevation might be enough for most COVID-19 patients for survival, particularly if the oxygen partial pressure of the inspired air is also elevated e.g., up to 30–40%. In conventional HBOT, (2.5 ATA, corresponding to a 15 m dive), the amount of blood plasma dissolved oxygen rises 20-fold (i.e., to 6 mL oxygen in 100 mL blood). 

Modern aircrafts resemble pressure chambers as they are usually pressurized to about 2400 m above sea level (from about 0.33 ATA/250 mmHg at 10,000 m to 0.76 ATA/577 mmHg at 2400 m) and the cabin pressure is thus elevated by about 0.45 ATA. The proposal to use some of several hundreds of ground-bound aircrafts in British airports to treat critically ill COVID-19 patients in 1.3–1.5 ATA elevated pressure was made in June 2020 by Dr. Philip James [80]. This proposal was made after reports from China, where oxygen treatment using HBOT when necessary was adopted in Wuhan at the end of March 2020 with dramatically good results [74]. In total, 4400 COVID-19-related deaths were reported in China up to March 2020; there have only been about 250 more to date.

In addition to reports from China, there are already multiple case-control studies reporting the use of HBOT for patients with COVID-19. All these studies state that HBOT is a safe and promising alternative for the treatment of COVID-19 patients. Some studies specifically describe patients reporting the prompt resolution of labored breathing following a single HBOT treatment [81,82,83,84,85]. In one very recent study, aimed at becoming RCT-compatible, it was possible to correct severe hypoxemia with mild HBOT (0.45 ATA overpressure) in three days compared with nine days in the controls [86]. The elevation of the pressure by 0.45 ATA (corresponding to an altitude of 2400 m) is equal to that used in airplanes prophylactically to correct potentially fatal hypobaria. Another recent study used conventional HBOT, 10 sessions at 2.4 ATA, to treat long COVID-19 patients with disabling fatigue and found statistically significant beneficial effects [87]. In a recent case report, HBOT was successfully applied to treat long COVID-19 symptoms, leading to improvements in cognition and cardiopulmonary function. This effect was proposed to be due to the ability of HBOT to reverse hypoxia, reduce neuroinflammation, and induce neuroplasticity [88].

Additional evidence of the potential usefulness of HBOT in brain hypoxia or ischemia comes from a large number of clinical studies demonstrating beneficial effects of HBOT. Similar to COVID-19, especially long COVID-19, which is characterized by reduced memory and cognitive functions in addition to tiredness, brain hypoxia has been proposed to be the underlying mechanism in such neurological diseases as Alzheimer’s disease, traumatic brain injury, cerebral palsy, and stroke (see Fisher and Barak 2020 for review) [89].

The most convincing evidence comes from management of stroke patients, where a considerable amount of preclinical research supports the post-stroke use of HBOT for damaged brain tissue. However, earlier controlled clinical trials of HBOT for stroke patients have yielded nonconclusive and somewhat contradicting results. This has changed along with a more recent breakthrough as prospective, randomized controlled studies have presented convincing evidence that HBOT can be the coveted neurotherapeutic method for brain repair [90,91,92,93]. 

The authors of COVID-19-HBOT studies conclude in common that well-defined RCT-compatible clinical trials are urgently needed [82,83,84,85,86]. However, designing such a trial may be difficult. Not only is it challenging to design a placebo treatment, but it might be even more difficult to randomize patients for the active vs. placebo treatment when the primary end point is death. HBOT has been accepted worldwide as the gold standard treatment for scuba-diving associated decompression (divers) disease, without any RCT evidence—because there also, the primary endpoint would be death. We propose the corresponding strategy—an elevated ambient pressure (1.3–1.5 ATA) for the treatment of COVID-19 patients with respiratory distress, using the higher pressures used in “conventional” HBOT (2.0–2.5 ATA) if necessary.

## 5. Reversing the ANS/CAN Imbalance in COVID-19 and Long COVID-19

Most of the early extrapulmonary symptoms of COVID-19 can be due to sympathetic dominance (and reduced parasympathetic function). Therefore, returning the ANS imbalance to normal by improving parasympathetic activity might be an important part of the therapeutic regimen. In addition, both the severe CNS and pulmonary disease are probably caused, and the pulmonary infection accentuated, through an inflammatory reflex mechanism due to inadequate immunological defense by the neuro-immune axis [94]. Excessive inflammation plays an important role in the pathogenesis of common and debilitating diseases, including septic shock [95].

### Inflammation and VNS

Although the common pathways between stress exposure and pathophysiological processes leading to tissue damage are still debatable, several results indicate that stress can activate an inflammatory response in the brain and in the periphery [96]. Stress-induced pro-inflammatory factors play an important role in this damaging process. In common, over-activated immune systems, increased sympathetic nervous system activity, and reduced glucocorticoid (GC) responsiveness may work in tandem in the activation of inflammatory responses during stress. As the vagal system with the vagus nerve in front is responsible for parasympathetic activity, neuromodulation via vagal nerve stimulation (VNS) can serve as a targeted treatment in stressful conditions. 

When the stimulation patterns and dynamics of functional networks during VNS were examined by fMRI, the vagus nerve was found to convey signals to the brain through the polysynaptic neuronal pathways by projecting to the brainstem nuclei (nucleus tractus solitarius, NTS, locus coeruleus), subcortical areas, and lastly, the cortex [97,98], thus covering the entire CAN. fMRI and a spatially independent component analysis were utilized in a recent experimental study [99]. That study demonstrated that VNS activated 15 out of 20 brain networks and that the activated regions covered >75% of the brain volume. There is strong preclinical scientific evidence for the beneficial role of VNS in the treatment of immunologic reflex-associated disorders, particularly rheumatoid arthritis (reviewed by Tracey, [100].

VNS has been conventionally performed for more than two decades to treat severe epilepsy and depression by applying an electrode surgically implanted into the cervical trunk of the vagus nerve. More recently, it has been shown by electrophysiological and neuroimaging studies that transcutaneous auricular VNS (taVNS) of the auricular branch of the vagus nerve (ABVN) activates central vagal pathways similar to the VNS with an implanted electrode [101,102].

In COVID-19/long COVID-19, taVNS may be especially effective, perhaps due to a dual action: it may attenuate the underlying neuroinflammation or inflammatory process in parallel or subsequent to stress response. As a method, taVNS is safer than VNS because the ABVN has no efferent neurons. In the pathogenesis of atrial fibrillation (AF), another common medical entity, accumulating evidence indicates that the inflammatory pathways play a significant role [103]. In a recent RCT-compatible clinical trial, chronic, intermittent taVNS resulted in significant reduction of inflammatory markers [104]. Based on its anti-inflammatory effects, taVNS targeted to the cervical part of the vagus nerve received emergency approval by the Federal Drug Administration in July 2020 for the treatment of asthmatic COVID-19 patients.

Earlier studies had failed to convincingly demonstrate that taVNS activates the crucial brainstem nuclei such as NTS. This has now changed: it was recently demonstrated using an ultrahigh-field (7T) fMRI that taVNS evokes activation in the ipsilateral NTS and upstream monoaminergic source nuclei of the brainstem [105]. Importantly, NTS activity is known to be modulated by respiration, both through the bottom-up afferent pathway from pulmonary stretch receptors and aortic baroreceptors and through the top-down effects from respiratory nuclei in the medulla [106]. Thus, taVNS treatment protocols should include instructions for slow breathing (“respiratory VNS”) [106]. 

The symptoms of “long COVID-19” are best explained by imbalance in the ANS, dysautonomia. This could be reversed by increasing parasympathetic activity, which can be done using various behavioral methods such as relaxation and breathing exercises (e.g., meditations) but more efficiently by weak electrical stimulation of the vagal system/nerve [107,108].

## 6. Brain Hypoxia May Be the Major Cause for COVID-19 and Long COVID-19 Symptoms

There is strong evidence that the symptomatology of both acute COVID-19 and long COVID-19 can best be explained by a pathophysiological mechanism in which brain hypoxia is a crucial component. Lack of oxygen in the brain, including the brain stem and medullary respiration centers, causes symptoms of acute severe COVID-19, and hypoperfusion-induced brain injury also manifests itself as symptoms of long COVID-19. This mechanism becomes obvious if the symptomatology is compared with the two best-known, most common conditions causing brain hypoxia, the carbon monoxide (CO) poisoning and hypobaric hypoxia (HH) due to altitude elevation. Small concentrations of CO in the inspired air cause headache and fatigue, symptoms that are also the first signal of brain hypoxia in mountain climbing as well as in aviation and ballooning. In mountain climbing, the first symptom of hypobaric hypoxia is headache. If the ascension is continued, the next symptoms will be fatigue and high-altitude pulmonary edema (HAPE). The next step is unconsciousness. HAPE is induced by a hypoxic environment [109,110] and is characterized by interstitial edema and a large amount of exudation in the pulmonary alveoli [111]. The frequently described “ground-glass opacity” in chest CTs of hospitalized COVID-19 patients also, at least if it occurs at the early stage of COVID-19, suggests HAPE-type pathogenesis and thereby may be a sign merely of hypoxia rather than of viral pneumonia. From HAPE and altitude medicine in general, it is known that even a small increase in pressure may be life-saving [78]. The symptoms of mild CO poisoning, HH, COVID-19, and long COVID-19 are listed in Table 2. It is obvious that the CNS-related symptoms of COVID-19 match perfectly with those of mild CO poisoning and HH.

Mild CO poisoning was also described by Haldane (1895) [112] as “the silent killer”, which brings to mind recent reports of “silent hypoxia” (and thereby a potential silent killer) in COVID-19. It is now also known that CO intoxication can cause, in survivors, serious brain damage into the midbrain the same as that seen in traumatic brain injuries. The mechanism of this was proposed already decades ago to be injury to the blood-brain-barrier (BBB) [124]. The BBB plays a crucial role in maintaining homeostasis within the CNS, and BBB breakdown is clearly evident in many neurological disorders. It has been shown in many recent studies. One recent in vitro study used a six-cell-type neurovascular unit human 3D organoid model containing brain microvascular endothelial cells, pericytes, astrocytes, oligodendrocytes, microglia, and neurons that recapitulates characteristics of BBB dysfunction under hypoxic physiological conditions to show that exposure to hypoxia results in BBB breakdown and subsequent its dysfunction [125]. Because the verification of brain damage usually remains undetected in clinical evaluations, COVID-19 patients with long-standing CNS-symptoms are often stigmatized as psychosomatic exaggerators or even simulants.

Thus, we feel strongly that in pathophysiological mechanisms of both acute and long COVID-19, early neuroinvasion of the virus is involved and that this leads to brain hypoperfusion and hypoxia. It has now been reported that long COVID-19 (in particular) has taught us to better understand the putative pathogenic mechanisms of such (for school medicine) “contested diseases” as POTS (postural orthostatic tachycardia syndrome), CFS (chronic fatigue syndrome), neuroborreliosis, and fibromyalgia. All these conditions are best explained by functional disturbances to the CAN [57], dysautonomia, and specifically the associated sympathetic dominance. Symptoms comparable with those of the present long COVID-19 were reported in long term follow-up studies of patients of the SARS 2002/3 epidemic in Toronto. We propose that the crucial component in the pathophysiologic mechanism of these “contested diseases” is the injury of BBB [124]. If that is true, it might teach us more about the pathogenetics of other presently enigmatic neurodegenerative diseases. 

## 7. Significance of Chest CT in COVID-19

Diagnosis of COVID-19 infection has largely been based on RT-PCR amplification of viral nucleic acid from the upper respiratory tract swabs. In addition, the main hallmark of COVID-19 pneumonia is the presence of ground-glass opacity (GGO) in lung computer tomography (CT). GGO, however, is not only a hallmark of pneumonia-type infection. It is merely a general sign of interstitial pulmonary edema occurring from diverse causes, of which the best characterized is the aforementioned HAPE from HH. Acute GGO has also been described as a serious complication in conditions such as during forceful swimming in triathlons and scuba diving and following electroconvulsive therapy (ECT), epileptic seizures, and CNS insults in general [126,127,128,129,130,131]. A characteristic feature of GGOs occurring in these connections is that they rapidly resolved with appropriate therapy, usually consisting of oxygen and diuretics. This may indicate that hypoxia is a component in such pathophysiologic GGO mechanisms because GGO in HAPE behaves similarly, resolving rapidly after only a relatively small descent. 

There are various theories concerning the pathogenetic mechanisms of GGO, again best studied in HAPE, using animal models. Although the results of animal studies cannot be directly applied to human, it is interesting that, according to such studies, the brain’s extremely complicated glymphatic systems are presented as playing a primary role, and in treatment aiming to resolve GGO, e.g., in rats, the positioning of animals, i.e., supine vs. sideways, plays an important role. Furthermore, there seem to be hitherto unknown mechanisms between the brain and the lungs manifesting in patients as GGOs after ECTs and various brain insults, named “neurogenic pulmonary edema” [131]. Abdennour et al. [132] even state that the brain and the lungs interact early and rapidly when hit by a disease process and furthermore that local brain inflammation spreads rapidly to the lung. 

It is generally held that the diagnosis of COVID-9 is based primarily on a combination of symptoms and positive results of virus PCR. In addition, chest CT plays a major role in the diagnostic workup, even if it is not recommended routinely in mild disease and it not infrequently shows GGO, which finding is interpreted as indicating severe disease. However, several reports suggest that GGO is relatively common in asymptomatic carriers of SARS-CoV-2. One of the first such reports, based on a multicenter study in China, performed chest-CT imaging for 411 symptomatic and 100 asymptomatic individuals, all PCR positive [133]. The surprise finding was GGO in 60% of asymptomatic individuals. About 25% of these later developed symptoms, suggesting GGO as the presymptomatic finding. Most of asymptomatic carriers with GGOs came from high-altitude areas, which supports the idea that (hypobaric) hypoxia might play a role in the development of GGO. Two other studies, consisting of 60 and 64 asymptomatic PCR positive carriers on whom chest CT had been performed, described GGO findings in 47 and 60%, respectively [134,135]. This caused De Smet et al. [134] to propose that in a pandemic setting, such incidental CT-GGO findings should be reported as “compatible with COVID-19 pneumonia” rather than as “viral pneumonia” and that non-infectious lung diseases should be excluded from the diagnosis. From a therapeutic viewpoint, it would be crucial to know whether early GGO in chest CT is a sign of viral pneumonia or of (brain) hypoxia.

## 8. Conclusions and Future Prospects

The COVID-19 pandemic is beginning to end, and novel therapeutic methods will not help many patients. For the future, however, it would be extremely important to learn the pathophysiological mechanisms of the disease and thereby design better treatments. It seems evident that the earliest symptoms of potentially severe COVID-19, including respiratory distress, are best explained by primary replication of SARS-CoV-2 in the nasal and/or olfactory epithelia, followed by invasion of the virus into the CNS, including the brainstem, and that ARDS is a later complication.

It seems plausible that SARS-CoV2 is neurotropic, first attacking the mucous membranes of the nasal cavity in the same way as the other six coronaviruses. From the nose, there is a short path to the brain, including the respiratory centers where the virus causes a mild infection or perhaps only injures the BBB. These lead to imbalance of the ANS and malfunction of the CAN with reduced parasympathetic (vagal) tone. The dysfunction of the vagal system can lead to inefficient respiration with subsequent hypoxemia and ultimately brain hypoxia that further worsens the efficacy of respiration. If the early, centrally triggered brain hypoxia were recognized, that would automatically lead to a change in therapeutic strategy from providing only supplemental oxygen to real oxygen therapy. Preliminary evidence on the use of elevated ambient pressure for COVID-19-associated respiratory failure is very promising. 

Several recent studies on long COVID-19 offer additional support for the idea that sympathovagal imbalance plays a crucial role. Therefore, VNS, or in practice taVNS, might offer a new, targeted therapeutic tool. Furthermore, taVNS is very patient-friendly and low-cost. However, as there are currently no appropriate online biomarkers available for taVNS, there is still a great need for additional research to find the optimal therapeutic regimen as well as better stimulating devices.

It will also be important that the medical community change its prevailing opinion and understand that oxygen itself is not toxic, that rather, with adequate dosing it has a crucial curative role. Many illnesses in which hypoxia may play an important pathogenetic role could be treated with pure oxygen and, if necessary, with elevated pressure. In the presence of a proven oxygen deficiency, it is not ethical to withhold additional oxygen, and at the correct dosage, which, as the Chinese studies have shown by measurements, may require the use of a pressure chamber. Although there is an enormous amount of reliable data on the importance of ambient pressure for the appropriate oxygenation of tissues, particularly for the most sensitive of them, the brain, it appears that the real significance of pressure in respiratory function is not fully understood even by most competent medical professionals. However, most of us may also not be aware that we have practically all been pressurized with 0.4–0.5 ATA and have breathed pressurized air in an airplane “hyperbaric chamber”. Airplane cabin pressurization is necessary (for survival) because atmospheric pressure falls during ascent (one millibar per 10 m). Correspondingly, the pressure increases when one descends from the sea level. The lowest place on earth is the Dead Sea, situated 457 m below sea level, which means that the average atmospheric pressure there is 802 mmHg. When a group of hypoxemic (COPD) patients (from Jerusalem, +850 m) stayed there for three weeks, subjective well-being and all measured functional parameters improved significantly [136]. Similar beneficial effects were also reported in patients with cystic fibrosis [137]. It seems likely that the improvement in tissue oxygen content using even compressed air at 1.3–1.5 ATA would be valuable judged on the well-established benefit of descent in high altitude pulmonary and cerebral edema. This pressure increase might be life-saving for many COVID-19 and other critically ill patients. If this alternative were included in the therapeutic regimen of COVID-19, it might be that this disease could be reduced in severity and become just another “flu”, i.e., influenza.

## Figures and Tables

**Table 1:** Atmospheric/ambient pressures under different baric conditions and corresponding oxygen tensions in alveoli, arteries, extracellular fluids of tissues, and mitochondria. ATA = atmospheres absolute; AP = atmospheric pressure; PO_2_ = partial pressure of oxygen; PAO_2_ = partial pressure of oxygen in alveoli; PaO_2_ = partial pressure of oxygen in arteries; PtO_2_ = partial pressure of oxygen in tissues; PmO_2_ = partial pressure of oxygen in mitochondria. * Estimations through extrapolations.

Atmospheric/Ambient		Oxygen Tension of Inspired Air, PO_2_	Oxygen Tension in Alvoli, PAO_2_	Oxygen Tension in Arteries, PaO_2_	Oxygen Tension in Tissues, PtO_2_	Oxygen Tension in Mitochondria, PmO_2_
Pressure (AP) mmHg		mmHg	mmHg	mmHg	mmHg	mmHg
2.5 ATA, AP 1875						
15 m diving, 100% O_2_ breathing		1875	1284 *	1274	250–500 [58,59]	80–125 *
		236	162 *	152	30–60 *	
5 m diving, 100% O_2_ breathing		1125	771 *	761 (59)	150–304 *	50–76 *
1.3 ATA, AP 988 air breathing		207	158 *	148 [60,61]	30–60 *	10–15 *
3 m diving, 100% O_2_ breathing		975	668 *	658	130–263 *	45–65 *
Sea level (1.0 ATA), AP 760 mmHg						
Air breathing [62,63,64] 20–50 y		160	102–110	97–99	20–40	7.5–11
	>64 y	-”-	-”-	82–93	16–37 *	
100% O_2_ breathing [63,64]		760	674	516	207 *	77 *
Dead Sea * −457 m AP 802 mmHg		167	114	104	42	15
Air						

**Table 2:** Lists of the most common CNS-related symptoms of mild CO poisoning, hypobaric hypoxia (HH), and COVID-19/long COVID-19. The symptoms of mild CO poisoning were listed in their order of prevalence by Haldane (1895). Order of prevalence was also intended by the authors in HH and COVID-19. Symptoms suggest hypoxia, primarily affecting the brain. In mountain climbing, the first symptom of HH is headache; the next are fatigue and high-altitude pulmonary edema (HAPE). The next step is unconsciousness. HAPE is induced by a hypoxic environment and is characterized by interstitial edema, shown as “ground-glass opacity” in chest CT.

Mild CO-Poisoning [112,113]	Aviation/Ballooning/Mountain Climbing	COVID-19/Long Covid
(in order of prevalence)	(Hypobaric hypoxia) [114,115,116,117]	[55,56,118,119,120,121,122,123]
Fatigue/lethargy	Visual disturbances	Anosmia
Headache	Headache	Fatigue
Numbness and tingling	Fatigue, lethargy	Headache
“Brain fog”	Dizziness, nausea	Dyspnea
Dizziness, nausea	Impaired fine touch & motor skills	“Brain fog”
Sleep disturbances	Personality & mood changes	Impaired consciousness
Palpitations	Sensory loss	Dizziness, nausea, tinnitus
Visual impairments	Confusion	Palpitations
Loss of consciousness	Loss of consciousness	Sleep disturbances
		Neuropsychological symptoms

## Data Availability

Not applicable.
